# Synthesis and Properties of Moisture-Cured Reactive Polyurethane Containing Castor Oil and Oxime Compounds

**DOI:** 10.3390/polym12081838

**Published:** 2020-08-17

**Authors:** Zheng-Ying Wu

**Affiliations:** Footwear & Recreation Technology Research Institute, No. 11, 8th Rd., Industrial Park, Taichung 407, Taiwan; wilsonwu389@hotmail.com or 0903@bestmotion.com

**Keywords:** castor oil, dimethylglyoxime, polyurethane, remeltable property, thermal property

## Abstract

Reactive polyurethane hot-melt resin (moisture-cured reactive polyurethane, PUR) could successfully be prepared from poly(tetramethylene ether) glycol (PTMG), castor oil and dimethylglyoxime (DMG) by one or two-stage synthesis. Fourier-transform infrared spectroscopy (FTIR) analysis showed that the synthesis resins belonged to NCO-capped castor oil-based polyurethane. The thermal behaviors of the cured PUR were analyzed by differential scanning calorimeter (DSC) and dynamic mechanical analyzer (DMA) instruments. The results showed that the cured resin provided remeltable properties under the dosages of 3 wt% DMG. Furthermore, the phenomenon could be proved by FTIR analysis according to the characteristic absorption peak of NCO groups after the cured resin was heated. Comparing different syntheses, the resin prepared by one-stage synthesis showed random distribution of DMG with PUR structure and that prepared by two-stage synthesis had distribution of DMG with branching structure in the prepolymer. The former obtained lower remeltable temperatures from 90 to 130 °C than the latter temperatures, which had temperatures above 125 °C. The tensile test showed that all of the PUR films exhibited typical tough behavior. Thus, the cured resin with DMG dosages of 3 wt% provided remeltable and mechanical properties at the same time. Overall, the crosslinking density and numbers of dynamic bonds should be kept in balance for preparation of remeltable PUR.

## 1. Introduction

Reactive polyurethane hot-melt resin (moisture-cured reactive polyurethane, PUR) is a non-solvent and NCO-capped prepolymer material. PUR is widely applied in films, foams, coatings and adhesives [1,2]. When PUR is exposed in the surroundings, the irreversible or non-meltable chemical crosslinking reaction goes through between the NCO of PUR and ambient moisture. Cross-linked PUR has excellent physical properties and other characteristics (e.g., chemical resistance, hydrolysis resistance and flexibility). However, the thermosets do not have reshaping, reprocessing or recycling ability. Recently, the design of polymer networks faces great challenges related to friendly polymers with excellent performance and recyclability. For this purpose, a large number of dynamic covalent bonds have been introduced into polymer networks. For the development of repairable crosslinked PUR the general method involves implanting dynamic bonds or redundant nucleophilic hydroxy groups on demand [3].

Polyols are the main raw materials for synthesis of PUR and the other primary materials are isocyanate compounds. All these materials are produced from petroleum-based compounds. To reduce dependence on petroleum resources, among them, polyols are the most likely to be replaced by biomass polyols derived from plants such as castor oil [4,5] and dimer acid-based derivatives [6]. Moreover, CO_2_-based polyols are another available resource [7]. Among them, castor oil (*Ricinus communis*, ricinoleic acid, s12-hydroxy-9-cis-octadecenoic acid) has hydroxyl groups in the chain without further derivatization, making it suitable for several applications in the synthesis of polyurethane [8,9,10].

In conclusion, the process of synthesizing PUR with castor oil and the properties of the remeltable chemical crosslinking reaction are bound to be interesting subjects. Therefore, for the purpose of simplifying the synthesis processes, we decided to prepare PUR containing castor oil and oxime compounds by one or two-stage synthesis and to evaluate the properties of PUR under various synthesis modes.

## 2. Materials and Methods

### 2.1. Materials

Poly(tetramethylene ether) glycol (PTMG, Mw = 1000), dimethylglyoxime (DMG), methylene diphenyl diisocyanate (MDI-50, MDI, Wanhua Chemical Group Co., Ltd., Yantai, China), castor oil (OH = 116.12, Young Sun Chemtrade Co., Ltd., Kaohsiung, Taiwan), dibutylamine (DBA) and hydrochloric acid (HCl). All these chemicals were used without further purification.

### 2.2. Synthesis of Moisture-Cured Reactive Polyurethane

The moisture-cured reactive polyurethane (reactive polyurethane hot-melt resin; PUR) with the formulations shown in Table 1 were prepared. All the polyols and DMG were heated in a vacuum oven at 60 °C until free of moisture. The polyols, PTMG and castor oil with weight of 100/20 were charged into a four-neck glass reaction flask. The dosage of MDI was calculated by the ratio of NCO_MDI_ to OH_(PTMG+Castor oil)_ in 2. Then, MDI weighing 63 g was added and polymerization was allowed to proceed with mixing at 100 °C under vacuum until the reaction was complete. The PUR product was the control. For the one-stage synthesis, DMG was used as a chain extender with dosages of 1.5 wt% and 3 wt%, based on the total weights of polyols and MDI, respectively. The polyols and DMG were added to the mix until the solution was homogeneous in the flask before polymerization. Then, synthesis of PU occurred after the MDI was added. For the two-stage synthesis, DMG was added after polymerization. The prepolymer reacted with the dosage of DMG. The resulting polymer was placed in a container under a dry nitrogen headspace to prevent exposure to moisture.

### 2.3. Preparation of Cured Reactive Polyurethane Film

All the PURs were shaped in a Teflon-coating box (length 20 cm, width 8 cm) and cured at 25 °C and below 60% relative humidity (RH). The thickness of film was about 0.40–0.50 mm.

### 2.4. Properties of Moisture-Cured Reactive Polyurethane

The viscosity of PUR was determined by a Brookfield viscometer (DV2T Viscometer, Brookfield, Middleboro, MA, USA). The measurements were performed using a spindle of NO. 27 at 20 rpm under 100 ± 1 °C by a heater. The NCO contents (NCO%) of PUR were determined according to ASTM D 2572-91. The resin was reacted with an excess of 0.5 N dibutylamine and then titrated with a standard 0.5 N HCl solution. The NCO values could be calculated.

### 2.5. FTIR Analysis

The infrared (IR) absorption spectra of the samples were measured using an FTIR spectrometer (PerkinElmer Spectrum 100, Waltham, MA, USA). The test was carried out by the single reflectance (single ATR) method and at the scanning range of 650–4000 cm^−1^.

### 2.6. DSC Thermo-Analysis

The DSC thermo-analysis was carried out by a differential scanning calorimeter (PerkinElmer DSC-7, Waltham, MA, USA) with the thermal-scanning method. The sample was heated from 25 to 180 °C at a heating rate of 10 °C/min. The variation in heat flow during the period of heat scanning was detected and the relative thermal parameters were calculated.

### 2.7. DMA Thermo-Analysis

The dynamic mechanical analyzer (DMA) thermo-analysis was carried out by a dynamic mechanical analyzer (Perkin-Elmer DMA 8000, Waltham, MA, USA). The thermal behavior of cured resins was investigated by the thermal-scanning method. The sample was heated from −80 to 160 °C at a heating rate of 5 °C/min and an oscillation frequency of 1.0 Hz by the tensile method.

### 2.8. TGA Thermo-Analysis

A thermal gravimetric analyzer (Perkin-Elmer Pyris 1 TGA-7, Waltham, MA, USA) was employed to measure the thermal degradation of cured resins. It was conducted under a nitrogen atmosphere by increasing the temperature from 50 to 700 °C at a heating rate of 10 °C/min.

### 2.9. Tensile Tests of Cured PUR Films

Tensile tests were carried out at 20 °C and 65% RH using a tensile testing machine (Shimadzu EZ Tester, Kyoto, Japan). The PUR films were conditioned for 48 h before the test. The sample was loaded at a crosshead rate of 10 mm/min.

## 3. Results and Discussion

### 3.1. Properties of PUR

**Figure 1:**
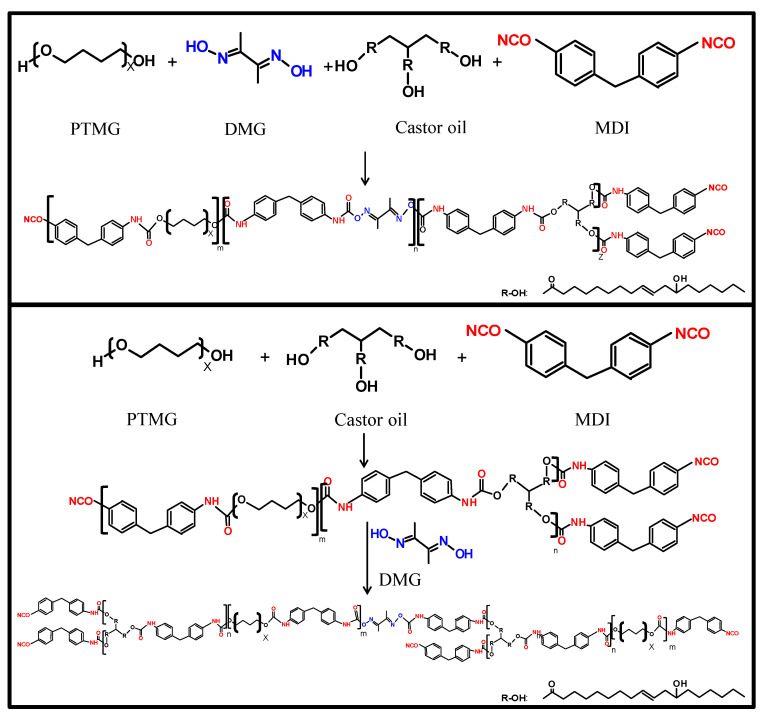
Reaction pathway of PUR containing castor oil and oxime compounds with various syntheses.

**Figure 2:**
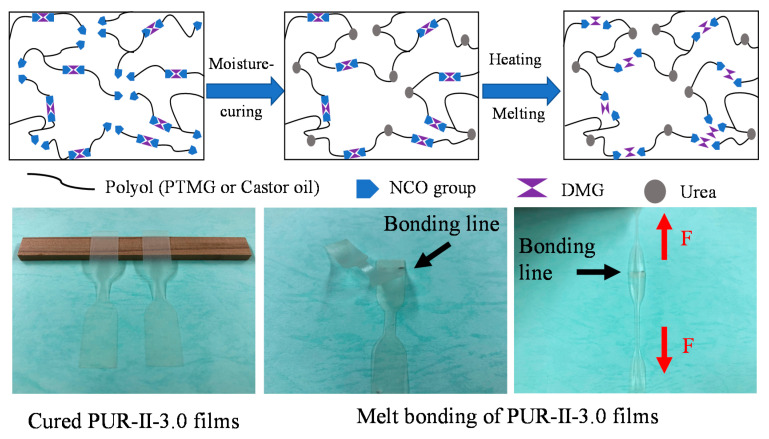
Curing, remeltable reaction and remeltable bonding of PUR containing castor oil and DMG.

PUR containing castor oil and oxime compounds were prepared from mixtures of PTMG, castor oil and MDI. The matrices of PUR were also implanted with DMG as an oxime compound by one or two-stage synthesis. The reaction pathway and curing reaction of PUR containing castor oil and DMG are shown in Figure 1 and Figure 2, respectively. In the one-stage synthesis, the raw materials, including PTMG, castor oil and DMG, reacted with MDI randomly under the condition of NCO/OH > 1. After that, the resin became an NCO-capped polyurethane. In the two-stage synthesis, the prepolymer synthesized from PTMG, castor oil and MDI first reacted with DMG. Basic properties of various PUR are shown in Table 1. Because of the implantment of DMG as a chain extender in PUR, the resins had less NCO groups and higher viscosity than the control. The viscosity of the resins increased when increasing the dosages of DMG, especially under the dosages of 3 wt% by two-stage synthesis. It was the presence of castor oil, due to its greater functionality, that led to significant branch structure in the resins prepared by two-stage synthesis.

Figure 2 showed that the implantment of DMG in PUR became the sites of dynamic covalent bonds. As the cured PUR containing DMG was heated, the urethane structures from DMG decomposed into NCO and OH groups. After that, the NCO groups generated would react with moisture or DMG in the resin. Thus, it could have remeltable property due to dynamic covalent bonds in polymer networks [3]. For the melt bonding test, the two cured films containing DMG were overlapped with the treatment of hot press. They could be bonded by heating.

### 3.2. FTIR of PUR

**Figure 3:**
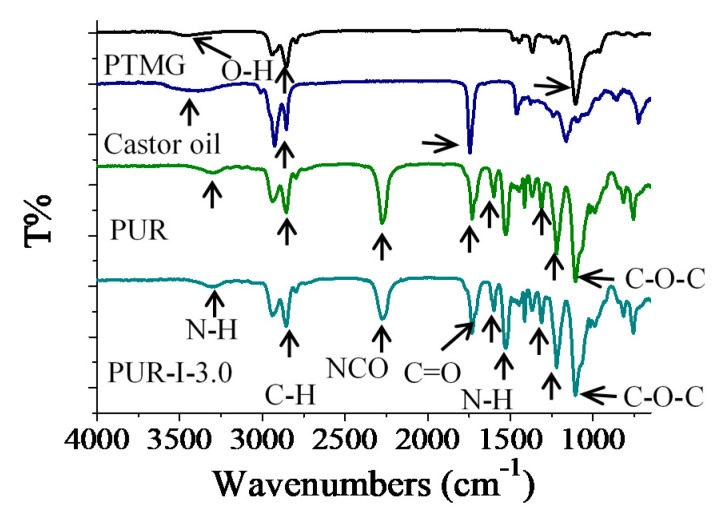
FTIR spectra of PTMG, castor oil and various cured PUR.

In this study, PTMG, castor oil and DMG were employed to react with the excess of MDI to prepare NCO-capped polyurethane. FTIR spectra of the raw materials and PUR are shown in Figure 3. As for the characteristic absorption peaks of PTMG, they were at 3200–3500 cm^−1^ for O–H stretching vibrations, 2700–3000 cm^−1^ for C–H asymmetric and symmetric stretching vibrations of methyl groups and 1100 cm^−1^ for C–O–C stretching (ether groups) in the structure. The FTIR spectra of castor oil was characterized with O–H and C–H stretching vibrations, with ester groups (C=O) in the structure at 1744 cm^−1^. In the spectra of the control and PUR-I-3.0, it was hard to distinguish the implantment of DMG. The spectra showed that the characteristic absorption peak of NCO groups was at 2722 cm^−1^. The bands at 3200–3400 cm^−1^, 1534 cm^−1^ and 1730 cm^−1^ were NH stretching vibrations, N–H for amide bands and ester groups (C=O) in urethane structure, respectively [11]. This illustrates that both resins were characterized as NCO-capped polyurethane. Most characteristic absorption peaks of PUR were close to those with PTMG serving as the main polyols. Moreover, the peak strength of C–H increased slightly due to the presence of castor oil.

**Figure 4:**
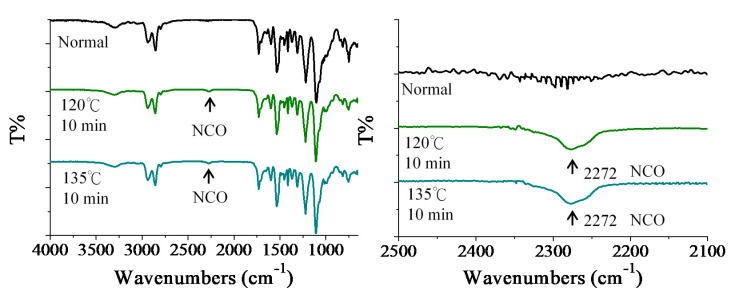
FTIR spectra of cured PUR-I-3.0 before and after heat treatment.

Figure 4 shows the FTIR spectra of cured PUR-I-3.0 before and after heat treatment. When the cured PUR-I-3.0 was heated at the temperature of 120 °C for 10 min or 135 °C for 10 min, respectively, the characteristic absorption peaks of NCO groups occurred in the spectra. This could explain why the dynamic bonds of PUR-I-3.0 had an effect with the implantment of DMG.

### 3.3. Thermal Behavior of Cured PUR

**Figure 5:**
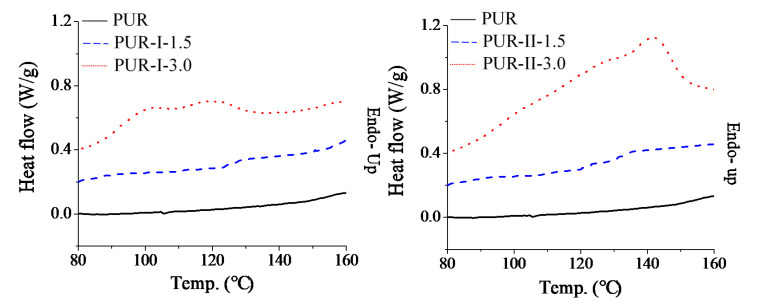
DSC thermal analysis of various cured PUR.

**Figure 6:**
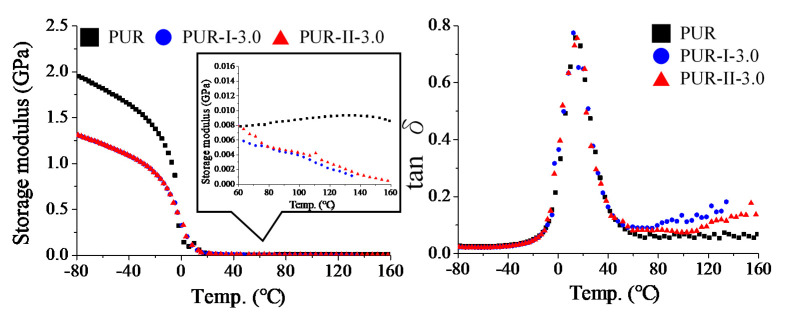
Dynamic mechanical analyzer (DMA) analysis of various cured PUR.

To realize the thermal behavior of the cured PUR, DSC and DMA thermal analyses were utilized to trace the heat flow and modulus of the resins by thermal scanning, as shown in Figure 5 and Figure 6, respectively. In DSC thermal analysis, the results showed that PUR-I-3.0 and PUR-II-3.0 had apparent endothermic heat flow, however, the control (PUR), PUR-I-1.5 and PUR-II-1.5, did not. This illustrates that crosslinking density and the numbers of dynamic bonds should be kept in balance to prepare remeltable PUR. In this case, the PUR with dosages of 3 wt% DMG could retain the remeltable property. However, the endothermic heat flow of cured PUR-I-3.0 was different from that of cured PUR-II-3.0. The former showed a broad curve of heat flow at lower temperatures from 90 to 130 °C. This resulted from the random distribution of DMG in the PUR structure in the one-stage synthesis. The latter showed a broad curve of heat flow at higher temperatures above 125 °C. This resulted from the distribution of DMG on the branch structure of the prepolymer.

Furthermore, the storage modulus and tan δ of the cured PUR were analyzed by DMA, as shown in Figure 6. For the control, its storage modulus was higher than PUR-I-3.0 and PUR-II-3.0 at negative temperature. The results illustrated the difference in mechanical strength. Thus, the control had a higher rigid mechanical property than PUR-I-3.0 and PUR-II-3.0 because of the higher crosslinking density. It was similar to the trend in the tensile tests of cured PUR films. For three resins, their moduli decreased with increasing temperature due to phase transition from glassy state to rubber state. From the tan δ curves of the cured resins, it showed that their glass transition temperature (Tg) was about 12 °C. Further, we observed the variation in storage moduli of the resins from 60 to 160 °C. The results revealed that the control did not have a remeltable property after being heated as a thermoset. As for PUR-I-3.0 and PUR-II-3.0, their storage moduli decreased with increasing temperature, dropping rapidly after the temperatures reached 90 °C and 110 °C, respectively. This phenomenon illustrated the remeltable property of cured resin. The storage modulus of PUR-II-3.0 was higher than that of PUR-I-3.0 after the temperatures reached 90 °C. The results could precisely illustrate the difference in the mechanical properties of the resins in the remelt state. It provided PUR-II-3.0 in the remelt state with a higher molecular due to the distribution of DMG on the branch structure of the prepolymer.

### 3.4. TGA Thermo-Analysis of Cured PUR

**Figure 7:**
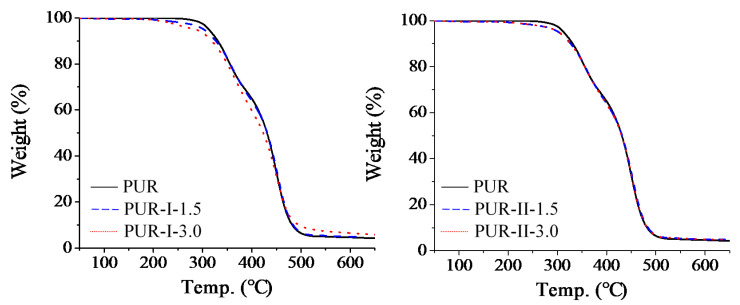
TGA curves of various cured PUR.

**Figure 8:**
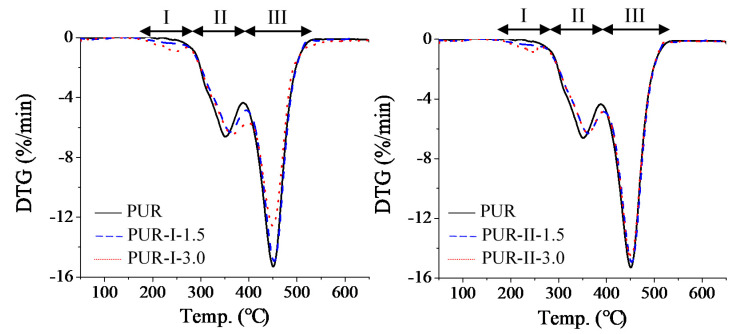
DTG curves of various cured PUR.

Figure 7 and Figure 8 show the TGA and DTG curves of the cured resins. Their TGA parameters are shown in Table 2. As shown by the TGA curves, the thermal degradation of the cured PUR occurred at temperatures of 180 °C to 500 °C. DTG analysis showed that the control had two stages of thermal degradation, the temperatures of which were 300 to 400 °C and 400 to 500 °C, respectively. However, all of the cured PUR containing DMG had lower temperatures of thermal degradation from 180 to 250 °C. This could be due to the decomposition of urethane linking with DMG. With increasing temperatures of 300 to 400 °C, decomposition of most urethane and urea bonds were observed [12,13]. The DTG peaks of the cured PUR containing DMG were similar to each other and shifted to higher temperatures than the control. This was caused by the decomposition of partial urethane groups during the early stage of thermal degradation. The heat resistance of the PU structure varied from high to low in the following order: cyanuric acid, urea, urethane, biuret and allophanate [14,15]. The stage from 400 to 500 °C was the fast dissociation phase of MDI, PTMG and castor oil chain scission [16,17]. All of the cured PUR had similar trends of thermal degradation with weight loss from 59% to 64%.

### 3.5. Mechanical Property of Cured PUR

**Figure 9:**
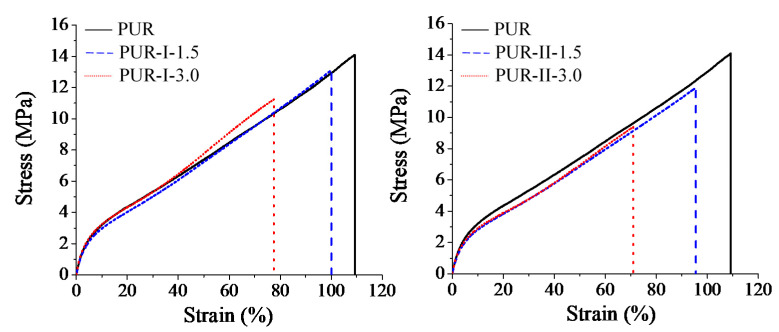
Stress–strain curves of various cured PUR.

Figure 9 shows the stress-strain curves of various PUR films. Table 2 summarizes the tensile strength, Young’s modulus and elongation at break of various cured PUR. The pure PUR film exhibited a typical tough behavior with tensile strength of 22.1 MPa and elongation at break of over 100%. For PUR containing DMG films, their tensile strength was from 20 to 25 MPa. Among them, the films with one-stage synthesis had more toughness. This was caused by the aggregation of the hard segment in the PU structure. Their elongation at break of 100% to 71% reduced as the dosage of DMG increased because the crosslinking density of films decreased.

## 4. Conclusions

The preparation of PUR containing castor oil and DMG by one or two-stage synthesis is feasible. According to FTIR, the thermal behavior and tensile tests of PUR, the cured resins with the dosages of 3 wt% DMG provided with remeltable and mechanical properties. Comparing different syntheses, the endothermic heat flow of cured PUR-I-3.0 appeared at lower temperatures from 90 to 130 °C than that of PUR-II-3.0, which appeared at temperatures above 125 °C. DMA analysis showed the same trend. Overall, for preparation of remeltable PUR, the crosslinking density and numbers of dynamic bonds should be kept in balance. The remeltable temperature of cured resin was affected by the distribution of DMG in the PUR structure under various syntheses.

## Figures and Tables

**Table 1:** Basic properties of various PUR.

Code of Resins	First-Step Synthesis				Second-Step Synthesis	NCO%	Viscosity (ps/100 °C)
	PTMG ^1^ (g)	Castor oil (g)	MDI ^1,2^ (g)	DMG ^1,3^ (wt%)	DMG ^1,3^ (wt%)		
PUR	100	20	63	-	-	5.73	71
PUR-I-1.5	100	20	63	1.5	-	4.60	150
PUR-I-3.0	100	20	63	3.0	-	3.48	252
PUR-II-1.5	100	20	63	-	1.5	4.56	181
PUR-II-3.0	100	20	63	-	3.0	3.41	320

^1^ PTMG: poly(tetramethylene ether) glycol (Mw = 1000); DMG: dimethylglyoxime; MDI: methylene diphenyl diisocyanate. ^2^ Calculated by the ratio of NCO_MDI_ to OH_(PTMG+Castor oil)_ in 2. ^3^ Based on the total weights of polyols and MDI.

**Table 2:** TGA parameters and mechanical properties of various PUR.

Code of Resins	TGA Parameters							Mechanical Properties		
	I Onset Temp. (°C)	I Weight Loss (%)	II Onset Temp. (°C)	II Weight Loss (%)	III Onset Temp. (°C)	III Weight Loss (%)	650 °C Residues (%)	Tensile Strength (MPa)	Young’s Modulus (MPa)	Elongation (%)
PUR	-	-	318.7	32.1	435.9	62.5	5.0	22.1	8.8	108.5
PUR-I-1.5	186.1	1.8	312.7	31.6	426.1	63.2	5.1	23.1	8.8	100.1
PUR-I-3.0	195.2	4.1	315.5	30.1	418.5	59.3	6.5	25.1	8.9	77.2
PUR-II-1.5	188.5	1.3	317.2	31.1	434.1	61.2	4.6	20.1	7.8	96.1
PUR-II-3.0	191.5	3.5	319.2	30.5	435.2	62.1	4.9	20.3	7.9	71.4
